# Communication of COVID-19 Misinformation on Social Media by Physicians in the US

**DOI:** 10.1001/jamanetworkopen.2023.28928

**Published:** 2023-08-15

**Authors:** Sahana Sule, Marisa C. DaCosta, Erin DeCou, Charlotte Gilson, Kate Wallace, Sarah L. Goff

**Affiliations:** 1 Department of Health Promotion and Policy, School of Public Health and Health Sciences, University of Massachusetts, Amherst

## Abstract

**Importance:**

Approximately one-third of the more than 1 100 000 confirmed COVID-19–related deaths as of January 18, 2023, were considered preventable if public health recommendations had been followed. Physicians’ propagation of misinformation about COVID-19 on social media and other internet-based platforms has raised professional, public health, and ethical concerns.

**Objective:**

To characterize (1) the types of COVID-19 misinformation propagated by US physicians after vaccines became available, (2) the online platforms used, and (3) the characteristics of the physicians spreading misinformation.

**Design, Setting, and Participants:**

Using US Centers for Disease Control and Prevention guidelines for the prevention and treatment of COVID-19 infection during the study window to define misinformation, structured searches of high-use social media platforms (Twitter, Facebook, Instagram, Parler, and YouTube) and news sources (*The New York Times*, National Public Radio) were conducted to identify COVID-19 misinformation communicated by US-based physicians between January 2021 and December 2022. Physicians’ state of licensure and medical specialty were identified. The number of followers for each physician on 4 major platforms was extracted to estimate reach and qualitative content analysis of the messages was performed.

**Main Outcomes and Measures:**

Outcome measures included categories of COVID-19 misinformation propagated, the number and traits of physicians engaged in misinformation propagation, and the type of online media channels used to propagate misinformation and potential reach.

**Results:**

The propagation of COVID-19 misinformation was attributed to 52 physicians in 28 different specialties across all regions of the country. General misinformation categories included vaccines, medication, masks, and other (ie, conspiracy theories). Forty-two physicians (80.8%) posted vaccine misinformation, 40 (76.9%) propagated information in more than 1 category, and 20 (38.5%) posted misinformation on 5 or more platforms. Major themes identified included (1) disputing vaccine safety and effectiveness, (2) promoting medical treatments lacking scientific evidence and/or US Food and Drug Administration approval, (3) disputing mask-wearing effectiveness, and (4) other (unsubstantiated claims, eg, virus origin, government lies, and other conspiracy theories).

**Conclusions and Relevance:**

In this mixed-methods study of US physician propagation of COVID-19 misinformation on social media, results suggest widespread, inaccurate, and potentially harmful assertions made by physicians across the country who represented a range of subspecialties. Further research is needed to assess the extent of the potential harms associated with physician propagation of misinformation, the motivations for these behaviors, and potential legal and professional recourse to improve accountability for misinformation propagation.

## Key Points

**Question:**

What types of COVID-19 misinformation have been propagated online by US physicians and through what channels?

**Findings:**

In this mixed-methods study of high-use social media platforms, physicians from across the US and representing a range of medical specialties were found to propagate COVID-19 misinformation about vaccines, treatments, and masks on large social media and other online platforms and that many had a wide reach based on number of followers.

**Meaning:**

This study’s findings suggest a need for rigorous evaluation of harm that may be caused by physicians, who hold a uniquely trusted position in society, propagating misinformation; ethical and legal guidelines for propagation of misinformation are needed.

## Teaser

This mixed-methods study investigates the types of COVID-19 misinformation that have been propagated through social media by US physicians.

## Introduction

As of May 11, 2023, an estimated 1 128 000 COVID-19 deaths had occurred in the US,^1^ and nearly 14% of people infected by the COVID-19 virus have experienced the post–COVID-19 condition.^2,3^ As of December 2022, estimated death rates for unvaccinated persons in the US were 271 per 100 000 compared with 82 per 100 000 for those fully vaccinated, yet only 69.2% of eligible people had received the full primary vaccine series, and 15.5% had received the bivalent booster.^1^ Vaccination rates have varied by region throughout the pandemic despite widespread availability, with southeastern states having lower full primary series rates (52%) compared with northeastern states (80%).^1^ Other preventive behaviors, such as mask wearing and social distancing, have varied similarly by geographic region.^4,5^

Individual health behaviors related to COVID-19 have been attributed to complex social phenomena, including inconsistent recommendations by government entities early in the pandemic, mistrust of the scientific community, political polarization, and unclear or incorrect guidance from other sources.^6,7,8^ COVID-19 misinformation, defined as false, inaccurate, or misleading information according to the best evidence available at the time, and disinformation, defined as having an intentionally malicious purpose, have been ubiquitous on social media, despite major platforms’ COVID-19 misinformation policies.^9^ Medical misinformation was propagated long before the COVID-19 pandemic,^10^ but the internet increases reach and speed of dissemination, potentially exacerbating misinformation consequences during an unparalleled public health threat that has killed more than 7 million people across the globe.^11,12,13^

COVID-19 misinformation has been spread by many people on social medial platforms,^14^ but misinformation spread by physicians may be particularly pernicious.^15^ Physicians are often considered credible sources of medical and public health information, increasing the potential negative impact of physician-initiated misinformation. The US Food and Drug Administration (FDA) and others have called for action to limit the potential harm of physician-propagated COVID-19 misinformation.^15,16^ Despite the rising concerns voiced in news articles and opinion pieces, physician-propagated COVID-19 misinformation and its associated outcomes remain understudied.

This study aimed to address this gap in knowledge by examining COVID-19 misinformation communicated on social media platforms and other online sources by US physicians after vaccines were made available. Understanding the extent of this phenomenon, its potential impact, and associated professional, ethical, and legal ramifications may help to better understand the role that physician-propagated COVID-19 misinformation may have played in preventable COVID-19 deaths and mistrust in institutions.

## Methods

### Overview

This mixed-methods study sought to characterize the (1) type of COVID-19 misinformation physicians communicated online between January 1, 2021, and May 1, 2022; (2) social media and other online platforms where misinformation appeared; and (3) characteristics of the physicians. Physician age, sex, and race and ethnicity were not available on social media or other online postings. A decision was made to not infer these data from pictures or other means to avoid potential bias and misclassification. We defined COVID-19 misinformation as assertions unsupported by or contradicting US Centers for Disease Control and Prevention (CDC) guidance on COVID-19 prevention and treatment during the period assessed or contradicting the existing state of scientific evidence for any topics not covered by the CDC (eTable in Supplement 1). We conservatively classified inaccurate information as misinformation rather than disinformation because the intent of the propagator cannot be objectively assessed. The University of Massachusetts Institutional Review Board determined that this study did not meet criteria for human participant research. This study followed the Standards for Reporting Qualitative Research (SRQR) reporting guidelines.

### Data Collection

First, we conducted structured searches of social media platforms and general web searches in late spring of 2022 to identify media containing COVID-19 misinformation attributed to US-based physicians, defined as using doctor of medicine (MD) or doctor of osteopathic medicine (DO) after their name and being licensed to practice medicine in the US at some time or never licensed but working in the US. The start date was selected in relation to the availability of the COVID-19 vaccines. Search terms included the following: “COVID,” “vaccine,” “doctor” or “physician,” “ineffective,” “pharmaceutical,” “medication,” “ivermectin,” “hydroxychloroquine,” and “purchase.” Search terms were refined based on initial searches to include “COVID misinformation,” “doctor” or “physician,” and/or “conspiracy theory.” Conspiracy theories were defined as communicating skepticism of all information that does not fit the theory, overinterpreting evidence that fits the theory, and/or evidence of internal inconsistency.^17^ The platforms searched were selected based on the volume of news articles, popularity, and searchability (Instagram, Twitter, YouTube, Facebook, Parler, TikTok, *The New York Times*, National Public Radio)^18^; if the findings on one platform indicated that another platform could have additional new data, it was added to the search list. Due to the large volume and repetitiveness of Tweets, Twitter searches focused initially on America’s Frontline Doctors’ Twitter profile because of the volume of COVID-19 misinformation in its Tweets,^19^ its large following, and the potential for physicians propagating misinformation to follow the page. Followers of the America’s Frontline Doctors’ page with an MD or DO in their header were traced on Twitter and other platforms as well. General internet searches using Google’s search engine were conducted to identify misinformation attributed to physicians in third party platforms, such as local news articles.

The following information was collected from each source: physician’s name, medical specialty, the state(s) in which they were currently or had been licensed, whether their license to practice was active, had lapsed, or been revoked based on state medical board site searches, when the misinformation was posted (if available), from what source it was found, and the number of followers the physician had (if the source was a social media platform). Misinformation was classified into the following categories: medication, vaccine, mask/distancing, and other unsubstantiated or false claims. After the initial searches were completed, the physicians’ names were searched on the social media platforms and through general online searches to identify misinformation they posted that may have been missed in the initial searches.

### Statistical Analysis

#### Quantitative

Descriptive statistics were used to quantify the types of misinformation, the frequency in which they appeared, the platforms on which they were found, and characteristics of the physicians identified (eg, specialty and state[s] in which the physician was licensed). We calculated the total, median, and IQR for the number of followers on platforms with the highest volume of users (Twitter, Facebook, YouTube, Instagram) using Stata software, version 17 (StataCorp).

#### Qualitative

We performed directed qualitative content analysis^20^ of the misinformation using a validated rapid qualitative analysis approach.^21^ The analytic team (S.S. and M.D.) populated a templated summary table with misinformation text extracted from each media platform. The team divided the physician list and generated a summary of the misinformation associated with each of the physicians. In the second step of this analytic process, each team member individually identified pertinent and common themes, subthemes, and supporting quotes for each. After this was done individually, the team met to discuss their findings and combine the findings into a final list of themes and subthemes. Considerations regarding reflexivity included that S.G. is a public health professor and physician, and M.D. and S.S. are aspiring physicians, which may have increased sensitivity to potential harms.

## Results

A total of 52 US physicians were identified as having communicated COVID-19 misinformation in the period assessed. All but 2 were or had been licensed to practice medicine in the US; the others were researchers. The 50 physicians who currently were or had been licensed represented 28 distinct medical specialties (3 of 50 had 2 different specialties; primary care was the most common overall [18 (36.0%)]) and they were licensed or working in 29 states across the US (Figure and Table 1). Forty-four of the 50 physicians (88.0%) held an active license in at least 1 state; 3 (6.0%) did not have an active license, 4 (8.0%) had had a license suspended or revoked, and 1 (2.0%) had active licenses in 2 states and revoked/suspended licenses in 2 other states. Nearly one-third (16 of 52) were affiliated with groups with a history of propagating medical misinformation, such as America’s Frontline Doctors. Specific types of misinformation included the following: (1) vaccines were unsafe and/or ineffective, (2) masks and/or social distancing did not decrease risk for contracting COVID-19, (3) medications for prevention or treatment were effective despite not having completed clinical trials or having been FDA approved, and (4) other (eg, conspiracy theories).

**Figure.**
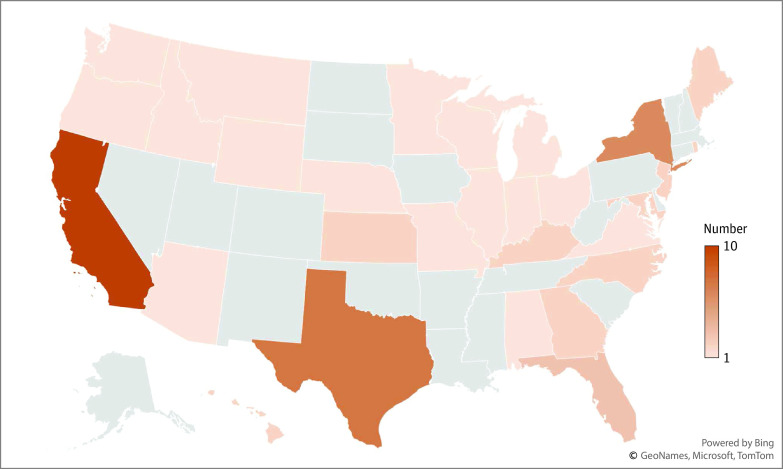
Distribution of Physicians Propagating Misinformation by State

**Table 1.** Physicians by Medical Specialty (N = 50)

Category and specialty	Physician No. (%)
Primary care	18 (36.0)
Family medicine	7 (14.0)
Internal medicine^a^	8 (16.0)
Pediatrics	1 (2.0)
Preventative medicine^a^	2 (4.0)
Medical subspecialty	12 (22.8)
Addiction medicine^a^	1 (2.0)
Allopathic medicine	1 (2.0)
Cardiac electrophysiology	1 (2.0)
Cardiology	2 (4.0)
Gastroenterology^a^	1 (2.0)
Hematology/oncology	1 (2.0)
Infectious disease	1 (2.0)
Integrative medicine^b^	1 (2.0)
Neurology^a^	1 (2.0)
Pulmonary critical care	2 (4.0)
Pediatric subspecialty	1 (2.0)
Pediatric cardiology	1 (2.0)
Surgical	8 (16.0)
Cardiac surgery	1 (2.0)
ENT	1 (2.0)
General surgery	1 (2.0)
GI surgery	1 (2.0)
Orthopedic surgery	1 (2.0)
OB-GYN	3 (6.0)
Other	14 (28.0)
Anesthesiology	1 (2.0)
Emergency medicine	2 (4.0)
Environmental medicine^a^	1 (2.0)
Ophthalmology	3 (6.0)
Pathology	1 (2.0)
Psychiatry	5 (10.0)
Radiology	1 (2.0)

^a^ A total of 3 of 50 physicians had 2 specialties.

^b^ A total of 16 additional physicians listed under other specialties offered services considered unconventional or integrative in their online information.

### Quantitative Analyses

Most of the 52 physicians (40 [76.9%]) who posted misinformation did so in more than 1 of the 4 categories identified. Vaccine misinformation was posted by the majority (42 [80.8%]), followed by other misinformation (28 [53.8%]; eg, government and public health officials deliberately falsified COVID-19 statistics) and medication misinformation (27 [51.9%]).

Of these 52 physicians, 20 (38.5%) posted COVID-19 misinformation on 5 or more different social media platforms and 40 (76.9%) appeared on 5 or more third-party online platforms such as news outlets. Twitter was the most used platform, with 37 of the 52 physicians (71.2%) posting misinformation and a median of 67 400 followers (IQR, 12 900-204 000). Additional details of physicians’ reach by platforms and followers are in Table 2 and Table 3.

**Table 2.** Physicians’ Reach by Number of Platforms

Physician reach	No. (%)
**No. of platforms posted on**	
>5	20 (38)
3-5	19 (37)
0-2	13 (25)
**No. of third-party platforms appeared on**	
>5	40 (77)
3-5	8 (15)
0-2	4 (8)

**Table 3.** Physicians’ Reach by Number of Followers

Most popular platforms by usage	No. of physicians (%)	Total followers	Median (IQR)
Twitter	37 (74)	9 137 588	67 400 (12 900-204 000)
Facebook	23 (44)	9 243 629	4122 (1925-27 000)
Instagram	19 (37)	2 567 971	11 000 (1608-131 000)
YouTube	5 (10)	214 862	24 700 (1570-41 100)

### Qualitative Analyses

Major themes identified included the following: (1) claiming vaccines were unsafe and/or ineffective, (2) promoting unapproved medications for prevention or treatment, (3) disputing mask-wearing effectiveness, and (4) other misinformation, including unsubstantiated claims, eg, virus origin, government lies, and other conspiracy theories. Supportive quotes are listed in Table 4.

**Table 4.** Supportive Quotations

Major themes and subthemes	Supportive quotations (platform, date, specialty)
Claiming vaccines were unsafe and/or ineffective	
Vaccine ineffectiveness	“It’s time to recognize natural immunity as at least as good as vaccination and end the mandates.” (Facebook, 2/4/22, anesthesiology)
	“One thing about natural immunity is that there has not been one reported case of someone getting reinfected and subsequently transmitting the virus to others.” (TikTok, 1/27/22, psychiatry)
	“The data are now abundantly clear. Natural imm [*sic*] is more effective than vax imm [*sic*].” (Twitter, 1/20/22, surgery)
	“61% of patients who tested positive at BreatheMD last month were vaccinated.” (Twitter, 2/26/22, ENT)
	“…recent data demonstrates that you are more likely to become infected or have disease or even death if you are vaccinated compared to unvaccinated people.” (TikTok, date unavailable, no specialty)
	“Why were there more COVID deaths in 2021 than 2020, even though we had vaccines in 2021 but not 2020?” (Twitter, 1/21/22, Psychiatry)
	“Question: if the COVID deaths are precipitously falling in several countries regardless of the % of the population vaccinated...why are we saying that the fall is due to vaccinations?” (Twitter, 2/27/22, OB-GYN)
	“Herd immunity dropped from propaganda campaign—became apparent mandated products: (1) do not stop transmission, (2) do not stop occurrence of URI, (3) are short-acting, (4) no health value to recovered. Even if everyone takes them—no impact on the pandemic.” (Facebook, 9/26/21, cardiology)
Vaccine risks	“According to the FDA’s adverse reporting system, 3 dozen cases of spontaneous miscarriages or stillbirths occurred after taking the COVID-19 vaccination. This raises ethical concerns about offering pregnant women experimental biological agents.” (Twitter, 3/8/21, emergency medicine)
	“VAERS data through the end of August 2022 shows that over 500 children have been permanently disabled as a result of the jab...” (Instagram, 9/10/21, integrative medicine)
	“...the chair of oncology at a large hospital in Florida said, ‘I usually see an aggressive brain cancer in a young patient about every decade or so and now I’ve seen five in the last month after the boosters...’” (TikTok, date unavailable, pathology)
	“Super safe and super effective. Especially in children (insert sarcastic eye roll). So safe that JAHA published two articles about jab induced myocarditis this month.” (Facebook, 10/31/21, family medicine)
	“Triple vaxxed are dying more than the other groups.” (Instagram, 3/5/22, ophthalmology)
	“Poor woman dies 7 minutes after her booster...while still in the drugstore. Article states that the coroner concluded that she died of natural causes. [clown emoji] When will this end?” (Facebook, 11/26/2022, critical care)
	“Most who took COVID Vaccines will be dead by 2025.” (YouTube, 10/20/21, preventive/environmental medicine/medical toxicology)
	“Of the 11,505 U.S. deaths reported as of March 4, 17% occurred within 24 hours of [COVID vaccination], 22% occurred within 48 hours, and 60% occurred in people who experienced an onset of injury symptoms within 48 hours of being taking it, suggesting there is some hope of saving lives.” (Twitter, 3/12/22, cardiology)
	“Get a vaccine. Get Sick.” (Facebook, 12/22/2021, psychiatry)
Promoting unapproved medications for prevention or treatment	
Anecdotal evidence of effectiveness	“A couple I knew came down with COVID-19….I recommended ivermectin, wrote a prescription for it. And again, you know it’s a [*sic*] off-label use, we use off-label drugs all the time as physicians. The FDA has not contraindicated ivermectin for the treatment of COVID-19. It does not have a black-box warning or anything, so it is ridiculous to make any issue of a physician prescribing ivermectin as an off-label use. Especially given the data from India and other countries in the world where ivermectin has been found quite effective in curtailing their COVID problems.” (YouTube, 10/20/21, anesthesiology)
	“Two of my toughest COVID patients—showed up with oxygen stats of 68% and 84% and would not go to the hospital. We treated them with IVM, steroids and breathing treatments and here they are now.” (Instagram, 5/6/21, ENT)
	“Ivermectin saved this patient and the dreaded HCQ. Wow look at that, personalized medicine at its best. #LETDRSBEDRS” (Twitter, 1/4/21, gastroenterology)
	“Attorney Ralph Lorigo has gone to court to force hospitals to give ivermectin to vented patients…12 times. He won court orders 11 times. 9 of those patients are now home and the 10th is rapidly improving on ivermectin. I guess this country needs more lawyers and less doctors.” (Twitter, 9/5/21, critical care)
Inaccurate claims of research-based “evidence” of effectiveness	“WHO’s ivermectin research team lead...independently publishes on 24 ivermectin RCT’s in a major journal—reports large decreases in mortality, hospitalization, time to recovery, viral clearance.” (Twitter, 7/8/21, critical care)
	“Head lice drug ivermectin is being explored as a potential treatment for the coronavirus following a promising new study that showed an 80% reduction in hospitalized COVID-19 patient deaths.” (Twitter retweet, 1/5/21, general surgery)
	“More data on IVM and doxycycline and effect on cytokine storms. Combined Therapy with Ivermectin and Doxycycline can effectively alleviate the Cytokine Storm of COVID-19 Infection and Vaccination Drive: A Narrative Review – ScienceDirect.” (Twitter, 4/5/21, gastroenterology)
	“MASSIVE peer reviewed study of Ivermectin concludes regular use as a prophylactic agent was associated with significantly reduced COVID-19 infection, hospitalization, and mortality rates.” (Twitter retweet, 3/8/21, OB-GYN)
Disputing mask-wearing effectiveness	
Ineffectiveness	“Since 2008 there have been 12 randomized controlled trial[s] about masking versus not masking to control respiratory viral infections and finally there was one that was done with covid itself and they share one thing in common, every single one of them has been negative.” (YouTube, 3/11/21, allopathic medicine)
	“Dozens of mask studies for decades have always shown they have little or no effect on viruses. Suddenly 2020 just ‘changed’ the science? Real scientists are skeptics—and this mask hysteria doesn’t add up.” (Twitter, 2/22/21, emergency medicine)
	“There’s really statistically no efficacy in masks.” (Insider, 9/15/21, pathology)
	“If we drop mask mandates too soon...The pandemic will take the exact same trajectory it otherwise would.” (Twitter, 2/13/21, hematology-oncology)
	“What if the experts are wrong?...What if wearing a mask in public is not effective?...I’m representing thousands of physicians across the country whose voices are being silenced because we don’t agree with the mainstream media and the experts who are telling us what to do.” (YouTube, 2/28/21, family medicine)
Negative consequences	“Remember this—if you wear a facemask you are restricting your oxygen, you are increasing the amount of carbon dioxide that you’re breathing back...” (YouTube, 7/14/20, preventive/environmental medicine/medical toxicology)
	“Study shows worse outcomes if you get covid because you are rebreathing the virus with a mask...” (TikTok, 7/25/22, psychiatry)
	“One of the most heartbreaking things about the pandemic has been what’s happening to the social interactions, the non-verbal cues...masks are not a natural thing for babies to have to interface with...” (YouTube, 3/25/21, family medicine)
	“Masking children in schools is child abuse. Those who demand you mask your kids are demanding child abuse. Masking kids eat outside in winter with masks on is child abuse. Call it what it is.” (Twitter retweet, 2/7/21, ophthalmology)
	“Forcing children to wear masks is child abuse. We must never allow this to happen again.” (Instagram, 3/28/21, OB-GYN)
Other misinformation	“What I found out was that this had been planned at least as far back as 2015, maybe even earlier, when it was realized that a plague of coronavirus could be created in the labs because they were doing...gain of lethal function research...” (YouTube, 5/26/21, psychiatry)
	“If this isn’t proof enough to convince the entire world that COVID was a planned operation, then we as a species are doomed...and probably deserve it.” (YouTube, 10/20/21, preventive/environmental medicine/medical toxicology)
	“…they [CDC] have made a strategic decision, which is an error in my opinion, to not be honest with the American people…” (TikTok, 9/6/21, neurology)
	“...I want a conversation with public health officials working for us about informing the nation on the pandemic. We cannot allow these practices to continue, they have done great damage to public health and have misled the nation.” (Twitter, 1/27/22, cardiology)
	“We need a death count audit immediately and accountability for these politicians who sold us a lie.” (Instagram, 2/7/21, family medicine)
	“Government actors across a dozen fed agencies were in contact with Twitter, with soc media, telling these social media companies what to censor and in many cases who to censor regarding COVID information.” (FOX News, 12/16/22, no specialty)
	“I knew of and had even met some of the people who were involved in the cover-up of COVID having a lab origin, and so when papers were published...from...what I would now call cover-up scientists and doctors...attempted to claim that there was no way COVID could have come from a lab...the reasons didn’t make sense...” (YouTube, 10/31/22, internal medicine)
	“What they did is they covered it up, and then...they made it so that anyone who questioned that theory, the idea of a lab-leak, was treated as a fringe scientist...a conspiracy theorist. When they knew full well that so many of the top virologists in the world believed that the lab leak was a real possibility. It is a pattern of behavior by them. Whenever they’ve been challenged, they’ve marginalized and demonized opposing scientists...” (FOX News, 1/30/22, no specialty)
	“...every wonder whether this anti-ivermectin onslaught on tv, in newspapers, medical journals, medical societies, health agencies...is to keep the market open for Pfizer and Merck’s oral anti-viral pills now rushing through pipeline?” (Twitter, 9/5/21, critical care)
	“The pharmaceutical companies had a lot to gain by suppressing cheap, easy, generic drugs that are available worldwide, in favor of new technologies that can make them billions of dollars.” (Twitter, 4/20/21, emergency medicine)

Abbreviations: CDC, US Centers for Disease Control and Prevention; ENT, ear, nose, and throat; FDA, US Food and Drug Administration; HCQ, hydroxychloroquine; IVM, ivermectin; JAHA, Journal of the American Heart Association; OB-GYN, obstetrics and gynecology; RCT, randomized clinical trial; URI, upper respiratory infection; WHO, World Health Organization.

#### Claiming Vaccines Were Unsafe and/or Ineffective

The most common theme identified was physicians discouraging the public from receiving COVID-19 vaccines. Promoting fear and distrust of the vaccine and reliance on “natural” immunity were common subthemes.

##### Vaccine Ineffectiveness

Some of the misinformation propagated by physicians claimed that COVID-19 vaccines were ineffective at preventing COVID-19 spread. A common approach included circulating counts of positive case rates by vaccination status, claiming that most positive cases were among vaccinated individuals. This claim is technically true but misleading, as many more people are vaccinated, and the proportion of unvaccinated people who are infected is much higher.^22^ Some stated that the significant increase in case rates after the initial vaccine rollout was evidence for ineffectiveness.

##### Vaccine Risks

Assertions that COVID-19 vaccines were harmful was not supported by scientific evidence at the time. Unfounded claims included that the vaccines caused infertility, irreparable damage to one’s immune system, increased risk of developing a chronic illness for children, and a higher risk of cancer and death. Claims that myocarditis was common in children who received the vaccine and that the risks of myocarditis outweighed the risk of vaccination were also unfounded.^23^ Several physicians redistributed news articles with stories of individuals suddenly or mysteriously dying from the vaccine, despite evidence from the CDC confirming that deaths caused by a COVID vaccine were extremely rare (9 deaths for over 600 million doses administered in the US as of January 2023) and could be attributed only to the Johnson and Johnson COVID-19 vaccine, which was used much less frequently than other manufacturers’ vaccines in many countries.^24^

#### Promoting Unapproved Medications for Prevention or Treatment

Many of the identified physicians promoted the use of treatments that had not been tested or FDA approved for use in relation to COVID-19. The 2 most prominent medications promoted were ivermectin and hydroxychloroquine, which have been found to not be effective at treating COVID-19 infections in randomized clinical trials.^25,26^

##### Anecdotal Evidence of Effectiveness

Anecdotal personal experiences of successfully treating patients with untested medications were commonly used to support claims about safety and effectiveness, such as patients’ conditions were not improving before receiving the untested medication, but the patient recovered after starting the treatment.

##### Inaccurate Claims of Research-Based Evidence of Effectiveness

Many physicians posted links or screenshots to articles claiming that ivermectin decreased mortality and hospitalization and increased time to recovery and viral clearance. Although some of the articles appeared to be peer-reviewed, none were in high-quality peer-reviewed biomedical journals, and the FDA had not approved the use of these medications for treating COVID-19. At least 1 of the cited articles has been retracted due to misinterpretation of the data.^27^

#### Disputing Mask-Wearing Effectiveness

Many of the physicians propagating misinformation about masking effectiveness portrayed masks in a negative light. Claims centered on ineffectiveness, harm, or both.

##### Ineffectiveness

Most of the misinformation propagated about wearing protective masks asserted that studies conducted before the pandemic definitively concluded that masks do not prevent the spread of respiratory viral infections. Additionally, data showing rising cases in areas enforcing mask mandates were interpreted to mean that the mandates did nothing to slow the spread of infection.

##### Negative Consequences

Allegations of consequences of mask wearing included medical and social or developmental effects, all of which were unfounded.^28^ Alleged medical consequences included claims that wearing a face mask restricts one’s oxygen, increases the amount of carbon dioxide being inhaled, and causes mask wearers to inhale bacteria that gets trapped. Many physicians focused on negative consequences related to children and mask mandates in schools, claiming that masks interfered with social development despite lack of evidence and that requiring children to wear masks was a form of child abuse.

#### Other Misinformation

This misinformation category included conspiracy theories related to domestic and foreign governments and pharmaceutical companies. Theories related to the government included the following: (1) the COVID-19 pandemic was planned by government officials—the “plandemic”; (2) government and public health officials withheld key information regarding COVID-19 from the public, such as hydroxychloroquine effectiveness, falsified statistics to make the virus appear more severe, and censored information that challenged government messaging; (3) the virus originated in a laboratory in China, which contradicted scientific evidence at the time; and (4) the virus was part of a National Institutes of Health–funded study, was leaked, and that the leak was covered up by government and public health officials. Theories related to pharmaceutical companies included that they played a role in discouraging the use of ivermectin and hydroxychloroquine because these medications were inexpensive and easily accessible, and pharmaceutical companies benefited from the promotion of more novel and expensive treatments.

## Discussion

This study was the first, to our knowledge, to identify the types of COVID-19 misinformation propagated by US physicians on social media and the platforms they used, as well as characterize the physicians who spread the misinformation. The content of misinformation physicians spread was similar to the misinformation spread by others; this study contributes new information about the range of specialties and regions of the country the physicians represented. The widely varying number of followers on social media for each physician suggested that the impact of any individual physician’s social media postings also may vary.

Some of the physicians identified belonged to organizations that have been propagating medical misinformation for decades,^10^ but these organizations became more vocal and visible in the context of the pandemic’s public health crisis, political divisiveness, and social isolation. Understanding the motivation for misinformation propagation is beyond the scope of this study, but it has become an increasingly profitable industry within and outside of medicine. For example, America’s Frontline Doctors implemented a telemedicine service that charged $90 per consult, primarily to prescribe hydroxychloroquine and ivermectin for COVID-19 to patients across the country, profiting at least $15 million from the endeavor.^29^ Twitter’s elimination of safeguards against misinformation^30^ and the absence of federal laws regulating medical misinformation on social media platforms suggest that misinformation about COVID-19 and other medical misinformation is likely to persist and may increase. Deregulation of COVID-19 misinformation on social media platforms may have far-reaching implications because consumers may struggle to evaluate the accuracy of the assertions made.^31^

National physicians’ organizations, such as the American Medical Association, have called for disciplinary action for physicians propagating COVID-19 misinformation,^32^ but stopping physicians from propagating COVID-19 misinformation outside of the patient encounter may be challenging.^33^ Although professional speech may be regulated by courts^34^ and the FDA has been called on to address medical misinformation,^16^ few physicians appear to have faced disciplinary action. Factors such as licensing boards’ lack of resources available to dedicate toward monitoring the internet^35^ and state government officials’ challenges to medical boards’ authority to discipline physicians propagating misinformation^36^ may limit action.

Scientific evidence depends on a body of accumulated research to inform practice and guidelines and the evidence depends on the best quality research available at any given time. A recent Cochrane Review has been misinterpreted to have definitively shown that wearing masks does not reduce transmission of respiratory viruses and has been used to support assertions that masks definitively “do not work.”^37^ Although the Federal Bureau of Investigation and Department of Energy presented a theory to Congress that the COVID-19 virus was the result of a laboratory leak,^38^ scientific evidence and a more recent report from the Office of the Director of National Intelligence demonstrate lack of evidence for a laboratory leak and favor a zoonotic origin of the virus.^39,40^ These recent challenges to prior understandings illuminate the importance of transparency and reproducibility of the process by which conclusions are drawn.

### Limitations

This study had some limitations. We conducted the study in the spring of 2022, after many major social media platforms had begun to establish policies to combat the propagation of COVID-19 misinformation, which means that the current study may underrepresent the extent of misinformation present before these policies were put in place. On some platforms (eg, Twitter), we were unable to analyze all posts by individuals due to the high volume of Tweets and degree of repetition. This study focused on online platforms whose content was readily accessible to the public; different approaches to identifying misinformation and searches of less used platforms might identify other physicians and include other topics. Misinformation disseminated in other ways, such as during clinical care, was not captured. Vaccines had been approved at the start of the period studied, but accessibility may have varied in the early days of the initial rollout. Finally, the state of scientific evidence for COVID-19 guidelines has evolved rapidly over the course of the pandemic, and this study represents a cross-section of time. The current evidence base for preventive and treatment practices, such as duration of vaccine effectiveness, may differ from the evidence base during the study time frame.

## Conclusions

Results of this mixed-methods study of the propagation of COVID-19 misinformation by US physicians on social media suggest that physician-propagated misinformation has reached many people during the pandemic and that physicians from a range of specialties and geographic regions have contributed to the “infodemic.” High-quality, ethical health care depends on inviolable trust between health care professionals, their patients, and society. Understanding the degree to which the misinformation about vaccines, medications, masks, and conspiracy theories spread by physicians on social media influences behaviors that put patients at risk for preventable harm, such as illness or death, will help to guide actions to regulate content or discipline physicians who participate in misinformation propagation related to COVID-19 or other conditions. A coordinated response by federal and state governments and the profession that takes free speech carefully into account is needed.

## Notes

Supplement 1. **eTable.** Misinformation vs Public Health Guidelines
eReferences

Supplement 2.
Data Sharing Statement
