# Errors in Quotes and Dates

**DOI:** 10.1001/jamanetworkopen.2023.41471

**Published:** 2023-10-25

In the Original Investigation, “Communication of COVID-19 Misinformation on Social Media by Physicians in the US,”^1^ published in *JAMA Network Open* on August 15, 2023, there were errors in Table 4. The errors included dates for 9 of the quotes, which had an incorrect year because of errors in coding the year as 2021 instead of 2022, but the months and days were correct as published. In addition, some quotes had very minor wording inaccuracies (eg, “One thing about natural immunity…” should be “One very important fact about natural immunity…”). The quote “…recent data demonstrates that you are more likely to become infected or have disease or even death if you are vaccinated compared to the unvaccinated people” should be “…recent data demonstrates that you are more likely to become infected or have disease or even death if you’ve been vaccinated compared to unvaccinated people.” The quote “I’m representing thousands of physicians” should be “I’m here representing thousands of physicians”; “…Government actors across a dozen fed agencies were in contact with Twitter, with soc media,” should be “…Government actors across a dozen federal agencies were in contact with Twitter, with social media”; “...every wonder whether” should be “...ever wonder whether”; and the word “the” was missing in “…now rushing through the pipeline.” A quote that was attributed to *Insider* should be *Business Insider*. Lastly, although the complete dates of the searches for this study, between January 2021 and December 2022, were included in the Abstract, the Methods section of the main article did not reiterate that the initial searches were extended through December 2022. This clarification has been added to the Methods section of the manuscript. Corrections for these errors do not affect the study findings, interpretations, or conclusions. The article has been corrected to address these errors.^1^
