# Supplementary material for: Communication of COVID-19 Misinformation on Social Media by Physicians in the US
Source: JAMA Netw Open. 2023 Aug 15;6(8):e2328928. doi: 10.1001/jamanetworkopen.2023.28928 (PMC10427940; doi:10.1001/jamanetworkopen.2023.28928)
Supplement: Supplement 1. — eTable. Misinformation vs Public Health Guidelines eReferences [file jamanetwopen-e2328928-s001.pdf]

Supplementary Online Content

Sule S, DaCosta MC, DeCou E, Gilson C, Wallace K, Goff SL. Communication of COVID-19 misinformation on social media by physicians in the US. *JAMA Netw Open*. 2023;6(8):e2328928. doi:10.1001/jamanetworkopen.2023.28928

eTable. Misinformation vs Public Health Guidelines

eReferences

This supplementary material has been provided by the authors to give readers additional information about their work.

© 2023 Sule S et al. *JAMA Network Open.*

eTable. Misinformation vs Public Health Guidelines

Misinformation Type	Public Health Guidelines
Vaccine ineffectiveness	• COVID-19 vaccines prevent serious illness, hospitalization, and death.[1]<br>• Building immunity through vaccination is safer and more reliable than getting infected.[1]
Vaccine risks	• The COVID-19 vaccines have been evaluated in several clinical trials and have met the FDAs rigorous scientific standards.[2]<br>• Adverse events and side effects are rare.[2]
Promotion of unapproved medications	• The benefits of hydroxychloroquine in treating COVID-19 do not outweigh the risks.[3]<br>• Ivermectin is not approved by the U.S. FDA to prevent or treat COVID-19 as there is insufficient data to prove its efficacy.[4]
Mask ineffectiveness	• Masking is a critical public health tool and reduces the spread of COVID-19.[5]
Mask risks	• Masks are made from breathable material that do not restrict oxygen flow nor trap carbon dioxide.[6]<br>• Masks have not been found to interfere with social development.[7]

© 2023 Sule S et al. *JAMA Network Open.*

eReferences

1. Benefits of Getting a COVID-19 Vaccine. Centers for Disease Control and Prevention. Published May 11, 2023. Accessed May 17, 2023. https://www.cdc.gov/coronavirus/2019-ncov/vaccines/vaccine-benefits.html

2. Safety of COVID-19 Vaccines. Centers for Disease Control and Prevention. Published March 7, 2023. Accessed May 19, 2023. https://www.cdc.gov/coronavirus/2019-ncov/vaccines/safety/safety-of-vaccines.html

3. Coronavirus disease (COVID-19): Hydroxychloroquine. Published March 28, 2023. Accessed May 17, 2023. https://www.who.int/news-room/questions-and-answers/item/coronavirus-disease-(covid-19)-hydroxychloroquine

4. Why You Should Not Use Ivermectin to Treat or Prevent COVID-19. *FDA*. Published online January 13, 2022. Accessed May 19, 2023. https://www.fda.gov/consumers/consumer-updates/why-you-should-not-use-ivermectin-treat-or-prevent-covid-19

5. Masks. Centers for Disease Control and Prevention. Published August 12, 2021. Accessed May 19, 2023. https://www.cdc.gov/coronavirus/2019-ncov/prevent-getting-sick/masks.html

6. Dickinson K, Guilbert T. Mask Mythbusters: Common Questions about Kids & Face Masks. HealthyChildren.org. Published March 24, 2022. Accessed May 17, 2023. https://healthychildren.org/English/health-issues/conditions/COVID-19/Pages/Mask-Mythbusters.aspx?_gl=1*1m222uc*_ga*MTc4OTQ1OTUxMy4xNjg0MzUwODgw*_ga_FD9D3XZVQQ*MTY4NDM1MDg4MC4xLjAuMTY4NDM1MDg4MC4wLjAuMA..&_ga=2.239303875.1551239792.1684350880-1789459513.1684350880

7. Yum LD. Do Masks Delay Speech and Language Development? CHC Resource Library | CHC | Services for Mental Health and Learning Differences for Young Children, Teens and Young Adults | Palo Alto, San Jose, Ravenswood. Published February 15, 2022. Accessed May 17, 2023. https://dev.chconline.org/resourcelibrary/do-masks-delay-speech-and-language-development/

© 2023 Sule S et al. *JAMA Network Open.*
