# Supplementary material for: Communication of COVID-19 Misinformation on Social Media by Physicians in the US
Source: JAMA Netw Open. 2023 Aug 15;6(8):e2328928. doi: 10.1001/jamanetworkopen.2023.28928 (PMC10427940; doi:10.1001/jamanetworkopen.2023.28928)
Supplement: Supplement 2. — Data Sharing Statement [file jamanetwopen-e2328928-s002.pdf]

Data Sharing Statement

Sule. Communication of COVID-19 Misinformation on Social Media by Physicians in the US. *JAMA Netw Open*. Published August 15, 2023. doi:10.1001/jamanetworkopen.2023.28928

Data
Data available: Yes
Data types: Data (not involving human participants)
How to access data: Data are all publicly available.
When available: With publication

Supporting Documents
Document types: None

Additional Information
Who can access the data: Researchers whose proposed use of the data has been approved.
Types of analyses: For a specified purpose.
Mechanisms of data availability: Without investigator support, after approval of proposal.
